# Disease burden and prognostic factors for clinical failure in elderly community acquired pneumonia patients

**DOI:** 10.1186/s12879-020-05362-3

**Published:** 2020-09-12

**Authors:** Xiudi Han, Xuedong Liu, Liang Chen, Yimin Wang, Hui Li, Fei Zhou, Xiqian Xing, Chunxiao Zhang, Lijun Suo, Jinxiang Wang, Guohua Yu, Guangqiang Wang, Xuexin Yao, Hongxia Yu, Lei Wang, Meng Liu, Chunxue Xue, Bo Liu, Xiaoli Zhu, Yanli Li, Ying Xiao, Xiaojing Cui, Lijuan Li, Bin Cao, for the CAP-China network

**Affiliations:** 1 Department of Respiratory Medicine, Qingdao Municipal Hospital Group, Jiaozhou Road, Qingdao City, 266011 Shandong Province China; 2 Department of Infectious Disease, Beijing Jishuitan Hospital, Xinjiekou East Street, Xi-cheng District, Beijing, 100044 China; 3 National Clinical Research Center of Respiratory Diseases,Center for Respiratory Diseases, China-Japan Friendship Hospital, Beijing, China; 4 Department of Pulmonary and Critical Care Medicine, China-Japan Friendship Hospital, Yinghuayuan East Street, Chao-yang District, Beijing, 100020 China; 5 Department of Respiratory Medicine, Yan’an Hospital Affiliated to Kunming Medical University, Renmin East Road, Kunming City, 652199 Yunnan Province China; 6 Department of Respiratory Medicine, Beijing Huimin Hospital, Youanmen Street, West District, Beijing, 100054 China; 7 Department of Respiratory Medicine, Linzi District People’s Hospital, Huangong Road, Zibo City, 255000 Shandong Province China; 8 Department of Respiratory Medicine, Beijing Luhe Hospital, Capital Medical University, Xinhua South Road, Tongzhou District, Beijing, 101149 China; 9 Department of Pulmonary and Critical Care Medicine, Weifang No. 2 People’s Hospital, Yuanxiao Street, Weifang City, 261599 Shandong Province China; 10 Department of Respiratory Medicine, Shandong University Affiliated Qilu Hospital (Qingdao), Hefei Road, Qingdao City, 266035 Shandong Province China; 11 Department of Respiratory Medicine, The 2nd Hospital of Beijing Corps, Chinese Armed Police Forces, Yuetan North Street, Xi-cheng District, Beijing, 100044 China; 12 Department of Infectious Disease, Qingdao University Medical College Affiliated Yantaiyuhuangding Hospital, Yudong Road, Yantai City, 100191 Shandong Province China; 13 Department of Respiratory Medicine, Rizhao Chinese Medical Hospital Affiliated to Shandong Chinese Medical University, Wanghai Road, Rizhao City, 276800 Shandong Province China; 14 Department of Respiratory Medicine, Beijing Hospital of Traditional Chinese Medicine Affiliated to Capital Medical University, Meishuguan Street, East District, Beijing, 100010 China; 15 Department of Occupational Medicine and Toxicology, Beijing Chao-Yang Hospital, Capital Medical University, Gongti South Road, Chao-yang District, Beijing, 100020 China

**Keywords:** Community-acquired pneumonia, Clinical failure, Treatment pattern, Elderly

## Abstract

**Background:**

The study was to evaluate initial antimicrobial regimen and clinical outcomes and to explore risk factors for clinical failure (CF) in elderly patients with community-acquired pneumonia (CAP).

**Methods:**

3011 hospitalized elderly patients were enrolled from 13 national teaching hospitals between January 1, 2014 and December 31, 2014 initiated by the CAP-China network. Risk factors for CF were screened by multivariable logistic regression analysis.

**Results:**

The incidence of CF in elderly CAP patients was 13.1%. CF patients were older, longer hospital stays and higher treatment costs than clinical success (CS) patients. The CF patients were more prone to present hyperglycemia, hyponatremia, hypoproteinemia, pleural effusion, respiratory failure and cardiovascular events. Inappropriate initial antimicrobial regimens in CF group were significantly higher than CS group. Undertreatment, CURB-65, PH < 7.3, PaO_2_/FiO_2_ < 200 mmHg, sodium < 130 mmol/L, healthcare-associated pneumonia, white blood cells > 10,000/mm^3^, pleural effusion and congestive heart failure were independent risk factors for CF in multivariable logistic regression analysis. Male and bronchiectasis were protective factors.

**Conclusions:**

Discordant therapy was a cause of CF. Early accurate detection and management of prevention to potential causes is likely to improve clinical outcomes in elderly patients CAP.

**Trial registration:**

A Retrospective Study on Hospitalized Patients With Community-acquired Pneumonia in China (CAP-China) (RSCAP-China), NCT02489578. Registered 16 March 2015, https://register.clinicaltrials.gov/prs/app/action/SelectProtocol?sid=S0005E5S&selectaction=Edit&uid=U0000GWC&ts=2&cx=1bnotb

## Background

Lower respiratory tract infections (LRTIs) fell to 4th place of global causes of deaths worldwide in 2016, yet community-acquired pneumonia (CAP) remains a leading cause of morbidity and mortality for hospitalization in LRTIs patients, especially for patients aged ≥65 years [1–3]. After initiation of empirical antibiotic therapy, patients with CAP can experience different clinical outcomes, clinical improvement, clinical failure (CF) and non-resolving pneumonia. CF is a matter of great concern in the management of CAP. The incidence of CF among hospitalized patients with CAP ranges from 6 to 24% [4–8], and can reach up to 31% for patients with severe CAP [9]. CF leads to longer hospital stay and higher mortality [4, 5, 9], longer duration of intravenous antimicrobial therapy [5], higher need for admission to intensive care unit (ICU) and more appearance of complications [4, 5].

Studies demonstrated that CF was due to an inadequate host–pathogen response, the initial severity of the infection, the presence of comorbidities, the causative organism, and the antimicrobial therapy administered [4, 5, 9]. Of which, underlying comorbidity [10, 11], severity of illness [10, 12] and antimicrobial regimen [13, 14] were independent factors for mortality of elderly hospitalized CAP. CF and mortality are the most relevant outcomes in elderly patients with CAP, yet there is rare discussion in literatures about its incidence and etiology.

It is crucial to understand the etiology of failure so as to implement different strategies at both national and local levels to prevent adverse outcomes. Therefore, we designed a retrospective study for hospitalized elderly CAP patients in order to identify the disease burden of CF and to explore the independent prognostic factors that predict failure to respond.

## Methods

### Study setting, design and participants

This study is a retrospective study utilizing patient and outcome data from the CAP-China network, which was a multicenter observational study of patients hospitalized with CAP. Data were abstracted from the database from January 1, 2014 to December 31, 2014 for all patients aged 65 years or older and included data from 13 centers in 7 cities of three provinces including Beijing, Shandong, and Yunnan (see Han et al. [15] for details concerning the patient cohort used in this study and the ‘Availability of Data and Materials’ section). The study was approved by the Human Subject Protection Program Institutional Review Board at China-Japan Friendship Hospital. Additional approval was obtained from the local internal review board for each participating hospital. Patient consent was waived due to the retrospective and observational study design. The initial antimicrobial regimen for each patient was evaluated and categorized as adherence, overtreatment, or undertreatment according to the 2016 Chinese CAP guidelines as previously described [15, 16]. Patients were stratified into ICU or ward admission status based on their initial admission orders.

### Definitions

CF was classified as early (≤72 h) or late (> 72 h). Early CF was defined as patients within the first 72 h of hospital admission: (1) progressive pneumonia if clinical deterioration with acute respiratory failure requiring ventilator support and/or septic shock appears; (2) non-responding pneumonia if persistence of fever and clinical symptoms without achieving clinical stability; and (3) death. Late CF was defined as patients after 72 h of initial antimicrobial treatment: (1) reappearance of fever with clinical deterioration (worsening of dyspnea with decrease of partial pressure of oxygen and/or increase of peripheral leukocyte count); (2) radiographic progression or the appearance of new infectious foci; (3) the need to switch to another antibiotic regimen resulting in an expansion of the antibiotic spectrum by adding another agent or replacing the initial antibiotic by another of the same class with a broader antibacterial spectrum and (4) death occurring after 30d of admission .

### Statistical analysis

The patients were divided into CF group and clinical success (CS) group. Data were presented as frequencies or percentages for categorical variables and the mean ± standard deviation for continuous variables. The characteristics of each group were compared using the Χ^2^ test for categorical variables and t test for continuous variables.

Variables showing significant difference in univariate analysis (*p* < 0.20) were included in multivariate Logistic analysis model for CF, and a stepwise forward model was used to select independent risk factors. The 95% confidence intervals (CIs) and level of significance were reported.

All data were analyzed with SPSS (version 20, IBM Corp., New York, USA); *p* < 0.05 was considered statistically significant.

## Results

### Incidence and baseline characteristics of clinical failure

A total of 3011 patients aged ≥65 years old were enrolled in final analysis. These following patients were excluded: (1) 62 patients of loss to follow-up; (2) 49 missing data on empirical antimicrobial regimens; (3)3 patients in the general ward who were administered antifungal agents and 6 patients in the ICU who were administered anti-pseudomonal β-lactam plus antifungal agents. CF occurred in 13.1% of elderly patients, 220 patients (55.7%) were early failure. Table 1 showed the demographic characteristics, comorbidities and initial severity of disease, as calculated by CURB-65 and PSI. Compared with CS group, CF patients were older, higher risk of aspiration, long-term bedridden confinement and *Pseudomonas aeruginosa* infection and more comorbidity. The rates of congestive heart failure (CHF), bronchiectasis, cerebral vascular disease and chronic renal disease were significantly higher in CF patients. The pneumonia severity in CF patients was more severe than that in CS patients, and more patients were admitted to ICU. Sixty patients (15.2%) reached to clinical stability on hospital admission, but still appeared failure.

**Table 1:** Comparison of baseline characteristics in hospitalized elderly CAP patients with clinical failure and clinical success (*n* = 3011)

Characteristics	CF(*n* = 395)	CS(*n* = 2616)	*p* value
Age (years)	79.3 ± 7.87	77.1 ± 7.3	<0.001
Male sex	209 (52.9)	1431 (54.7)	0.516
Aspiration	79 (20.0)	234 (8.9)	<0.001
Long-term bedridden status	74 (18.7)	185 (7.1)	<0.001
Underlying conditions	381 (96.5)	2344 (89.6)	<0.001
Cardiovascular disease	268 (67.8)	1607 (61.4)	0.014
Hypertension	215 (54.4)	1262 (48.2)	0.023
Ischemic heart disease	144 (36.5)	826 (31.6)	0.057
Congestive heart failure	46 (11.6)	150 (5.7)	<0.001
Chronic respiratory disease	106 (26.8)	735 (28.1)	0.631
COPD	77 (18.5)	482 (18.4)	0.627
Bronchiectasis	17 (4.3)	273 (10.4)	<0.001
Asthma	16 (4.1)	139 (5.3)	0.330
Cerebral vascular disease	141 (35.7)	641 (24.5)	<0.001
Diabetes mellitus	96 (24.3)	536 (20.5)	0.085
Malignancy	35 (8.9)	205 (7.8)	0.485
Chronic renal disease	37 (9.4)	114 (4.4)	<0.001
Dementia	11 (2.8)	44 (1.7)	0.154
Chronic liver disease	5 (1.3)	33 (1.3)	1.000
History of CAP within 1 year	52 (13.2)	207 (7.9)	0.001
HCAP	135 (34.2)	385 (14.7)	<0.001
Immunocompromise^#^	18 (4.6)	52 (2.0)	0.004
Numbers of comorbidities	2.4 ± 1.4	1.9 ± 1.3	<0.001
Severity on hospital admission			
CURB-65^a^	2.1 ± 0.9	1.5 ± 0.7	<0.001
PSI^b^	118.0 ± 32.2	88.9 ± 22.0	<0.001
ICU admission	147 (37.2)	102 (3.9)	<0.001
Outcomes			
Clinical stability on hospital admission	60 (15.2)	1015 (46.4)	<0.001
Hospital LOS (days)	16.4 ± 17.0	13.0 ± 8.2	<0.001
Total cost (RMB, median)	23,083.1	12,287.5	<0.001

*Abbreviations*: *CF* Clinical failure; *CS* Clinical success; *COPD* Chronic obstructive pulmonary disease; *CAP* Community acquired pneumonia; *HCAP* Healthcare-associated pneumonia; *PSI* Pneumonia severity index; *ICU* Intensive care unit; *LOS* Length of stay

^#^definition seen as Han et al. [15]

^a^ Figure urea nitrogen was missing in 124 cases. *n* = 2887

^b^ The total number of patients with a complete data of PSI score. *n* = 1666

### Clinical manifestations, laboratory and radiologic findings of clinical failure

Compared to CS group, Table 2 showed the rates of wheezing, cyanosis and lower extremity edema were more common in CF group, as well as unstable vital signs. Hyperglycemia, azotemia, hyponatremia, hypoproteinemia, acidosis and pleural effusion were more prone to occur in CF patients. During the whole progress of hospitalization, the incidence of respiratory failure, acute cardiovascular events and acute renal failure was notably different between two groups.

**Table 2:** Comparison of clinical manifestations, laboratory and radiologic findings in hospitalized elderly CAP patients with clinical failure and clinical success (*n* = 3011)

Characteristics	CF(*n* = 395)	CS(*n* = 2616)	*p* value
Clinical features			
Fever	208 (52.7)	1364 (52.1)	0.871
Cough	327 (82.8)	2368 (90.5)	<0.001
Chest pain	20 (5.1)	244 (9.3)	0.004
Wheezing	214 (54.2)	1034 (39.5)	<0.001
Cyanosis	89 (22.5)	292 (11.2)	<0.001
Lower extremity edema	98 (24.8)	366 (14.0)	<0.001
Mental confusion	2016 (26.8)	137 (5.2)	<0.001
RR > 24beats/min	62 (15.7)	119 (4.5)	<0.001
HR > 125beats/min	34 (8.6)	40 (1.5)	<0.001
Hypotension	92 (23.3)	386 (14.8)	<0.001
Laboratory findings			
WBC > 10,000/mm^3^(*n* = 2944)	172 (45.1)	762 (29.7)	<0.001
The rate of NE(*n* = 2917)	0.8 ± 0.2	0.7 ± 0.1	<0.001
The rate of L(*n* = 2890)	0.1 ± 0.1	0.2 ± 0.6	0.007
NE/L(*n* = 2889)	14.3 ± 16.5	7.1 ± 9.4	<0.001
HCT<30%(*n* = 2874)	86 (22.9)	310 (12.4)	<0.001
Cr > 123.76 umol/L (*n* = 2878)	68 (18.2)	217 (8.7)	<0.001
Glucose> 14 mmol/L(*n* = 2757)	19 (5.5)	68 (2.8)	0.013
Na<130 mmol/L(*n* = 2890)	55 (14.7)	141 (5.6)	<0.001
BUN> 7 mmol/L(*n* = 2887)	202 (53.6)	689 (27.5)	<0.001
PH<7.3(*n* = 1884)	24 (7.7)	21 (1.3)	<0.001
PaO_2_/FiO_2_<200 mmHg(*n* = 1857)	70 (23.0)	138 (5.9)	<0.001
Albumin<25 g/L(*n* = 2863)	57 (15.7)	154 (6.2)	<0.001
Complications			
Respiratory failure	329 (83.3)	196 (7.5)	<0.001
Arrhythmia	60 (15.2)	45 (1.7)	<0.001
Acute heart failure	151 (38.2)	199 (7.6)	<0.001
Acute myocardial infarction	24 (6.1)	9 (0.3)	<0.001
Acute liver failure	27 (6.8)	17 (0.6)	<0.001
Acute renal failure	63 (15.9)	14 (0.5)	<0.001
Stroke	17 (4.3)	15 (0.6)	<0.001
DIC	4 (1.0)	1 (0)	0.001
Gastrointestinal bleeding	47 (11.9)	21 (0.8)	<0.001
Thromboembolic disease	4 (1.0)	13 (0.5)	0.266
Radiology findings on CT			
Multilobe infiltration (*n* = 3011)	193 (48.9)	1181 (45.1)	0.178
Pleural effusion (*n* = 3011)	146 (37.0)	603 (23.1)	<0.001

*Abbreviations*: *RR* Respiratory rate; *HR* Heart rate; *WBC* White blood cell; *NE* Neutrophil; *L* Lymphocyte; *HCT* Hematocrit; *Cr* Creatinine; *Na* Sodium; *BUN* Blood urea nitrogen; *PaO*_*2*_*/FiO*_*2*,_ Partial arterial oxygen pressure/fraction of inspired oxygen; *DIC* Disseminated intravascular coagulation; *CT* Computed tomography

### Pathogens

Isolation of pathogens was defined in 73 (18.5%) patients of CF group; 54 (74%) patients isolated the gram-negative bacilli, and the top three pathogens were *Pseudomonas aeruginosa* (24.7%, 18/73), *Klebsiella pneumonia* (16.4%, 12/73) and *Acinetobacter* (13.7%, 10/73). 17(23.3%) patients isolated the gram-positive cocci. *Staphylococcus aureus* (15.1%, 11/73) was the most common pathogen, of which MRSA accounted for 90.9%(*n* = 7). *Streptococcus pneumoniae* was isolated in only 2 patients and no atypical pathogen was found. 9.6%(n = 7) patients were isolated influenza virus and 19.2%(*n* = 14) patients were mixed infections.

### Antimicrobial regimens

Compared to CS group, the rate of inappropriate antimicrobial regimens was significantly higher in CF patients(*p*<0.001). Table 3 showed that less than 30% of patients in CF group received initial treatment consistent with 2016 CAP guideline. Monotherapy with β-lactam in general ward or fluoroquinolone plus β-lactam in ICU was the most common regimen. The proportion of undertreatment and overtreatment was comparable in CF group. Regardless of CF group or CS group, the administration of anti-pseudomonal β-lactam accounted for a certain proportion.

**Table 3:** Comparison of antimicrobial regimens in hospitalized elderly CAP patients with clinical failure and clinical success (*n* = 3011)

Regimen	CF(*n* = 395)	CS(*n* = 2616)	*p* value
Consistent with guideline	115 (29.1)	1032 (39.4)	<0.001
β-lactam	76 (19.2)	579 (22.1)	
Fluoroquinolone	22 (5.6)	368 (14.1)	
β-lactam + macrolide	6 (1.5)	74 (2.8)	
β-lactam + fluoroquinolone	22 (5.6)	11 (0.4)	
Undertreated by guideline	138 (34.9)	396 (15.1)	<0.001
β-lactam	99 (25.1)	302 (11.5)	
β-lactam + macrolide	8 (2.0)	42 (1.6)	
Macrolide	1 (0.3)	18 (0.7)	
Fluoroquinolone +/− β-lactam/other	18 (4.6)	17 (0.6)	
Other combination	12 (3.0)	17 (0.6)	
Overtreated by guideline	142 (35.9)	1188 (45.4)	<0.001
Antipseudomonal β-lactam	47 (11.9)	468 (17.9)	
Fluoroquinolone +β-lactam/ other	71 (18.0)	585 (22.4)	
Antipseudomonal β-lactam+ macrolide	6 (1.5)	45 (1.7)	
Antipseudomonal β-lactam + other	11 (2.8)	52 (2.0)	
Fluoroquinolone + macrolide	0 (0)	17 (0.6)	
β-lactam +quinolone+ other	5 (1.3)	11 (0.4)	
β-lactam +macrolide+ fluoroquinolone /other	2 (0.5)	10 (0.4)	

*Note*: other = imidazoles, lincomycin, etracyclines, aminoglycoside, fosfomycin, glycopeptides and antifungal agents

### Outcomes

A total of 167 (5.5%) patients died during hospitalization, of which 41.9%(*n* = 70) of patients died of early CF. 221 (7.3%) died 30 days after discharge, although 35.7% (*n* = 1075) of patients reached clinical stability on hospital admission. The mortality rate and 30d mortality rate was 11.6% (*n* = 46) vs 16.7% (*n* = 66), 14.1%(*n* = 56) vs 18.5% (*n* = 73), 16.5%(*n* = 65) vs 20.8% (*n* = 82), respectively, in patients with treatment concordant to guideline, undertreatment and overtreatment in CF group.

Median overall treatment cost for one CAP episode was RMB 12,950.9 (mean ± SD RMB 24,564.7 ± 111,003.3). Treatment costs in successfully treated patients were below the overall median; however, they were nearly two-fold higher in failures (p<0.001). In CF group, median overall treatment cost for concordant patients was RMB 16,417.4 (mean ± SD RMB 46,714.9 ± 96,721.8), slightly lower than that in overtreated patients, RMB 22348.2 (mean ± SD RMB 47127.4 ± 63,894.2), but significantly lower than that of undertreated patients, RMB 34098.5 (mean ± SD RMB 70071.8 ± 1,033,803.9) (*p* = 0.039). The median antimicrobial costs were RMB 2742.9, RMB 3624.3 and RMB 5365.1 in patients with treatment concordant to guideline, undertreatment and overtreatment of CF group (*p* = 0.033). The median examination and hospitalization costs due to undertreatment were notably higher than concordant and overtreated patients (*p*<0.02).

### Predictors related with clinical failure

**Fig. 1:**
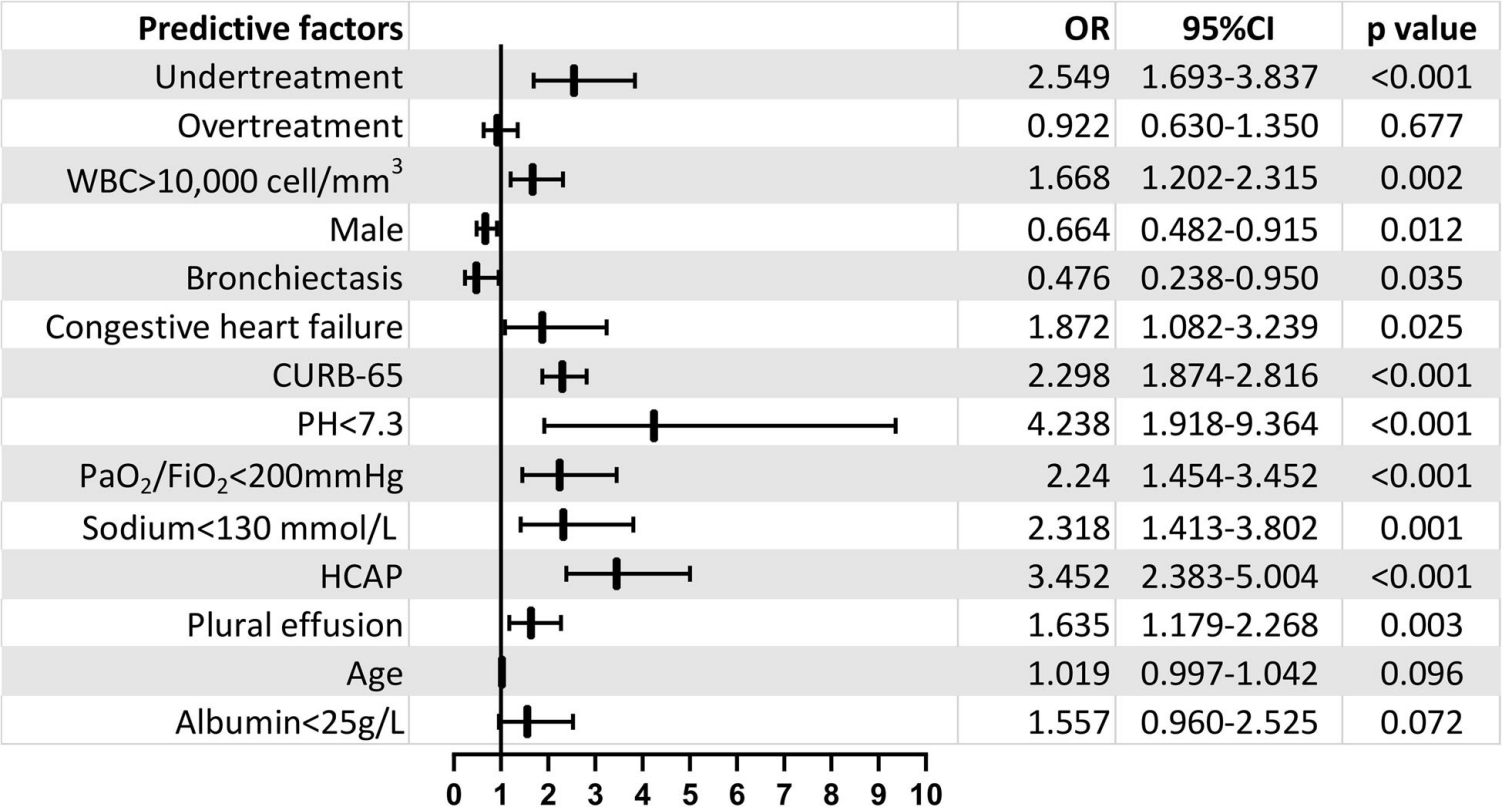
Multivariable logistic regression analysis of prognostic factors for clinical failure associated with CAP. WBC = white blood cell; HCAP = healthcare-associated pneumonia; OR = odds ratio; CI = confidence interval

The multivariable Logistic model showed undertreatment (*p* < 0.001), CURB-65(*p* < 0.001), PH<7.3(*p* < 0.001), PaO_2_/FiO_2_<200 mmHg (*p* < 0.001), Na^+^<130 mmol/L (*p* = 0.001), healthcare-associated pneumonia (HCAP) (*p* < 0.001), white blood cells (WBC) > 10,000/mm^3^ (*p* = 0.002), plural effusion(*p* = 0.003) and CHF (*p* = 0.025) were independent predictors for CF related with elderly CAP patients. While male (*p* = 0.012) and bronchiectasis (*p* = 0.035) were protective factors (seen in Fig. 1).

## Discussion

This is the first retrospective multicenter study to assess the disease burden of clinical failure in hospitalized elderly patients with CAP in China. The major findings of our study were as follows: 1) CF contributes to a significantly prolonged length of stay (LOS) and increased median overall treatment costs; 2) Independent risk factors for CF were undertreatment, high CURB-65 score, lower PH, lower PaO_2_/FiO_2_, hyponatremia, HCAP, leukocytosis, plural effusion and CHF; 3) Male and patients with bronchiectasis were associated with lower CF rate.

In our study, we adopted the term of clinical failure to include all patients whose condition deteriorated [9, 17]. Although data from a prospective series of 1383 non-immunosuppressed hospitalized adults with CAP demonstrated that older age (> 65 years) was an independent factor associated with clinical failure [5], the incidence of clinical failure in elderly hospitalized CAP patients in our study was 13.1%, lower than that in previous studies [4, 5, 8, 9, 18, 19], mainly due to larger proportion of mild-to-moderate patients in our study.

The median overall treatment cost for one CAP episode was RMB 12,950.9 (mean ± SD RMB 24,564.7 ± 111,003.3). And they were nearly two-fold higher in CF patients, which was in accordance with previously published data [19]. Furthermore, we found median overall treatment cost in undertreated patients of CF group was significantly higher, which was mainly relative to extra examination and prolonged hospital stay. Patients experiencing CF required a significantly prolonged hospital stay compared with successfully treated patients (mean LOS 16.4 ± 17.0 versus 13.0 ± 8.2 days), which was similar to other studies from Switzerland [8], Netherlands [9] and Germany [19]. Prolonged hospital stay was associated with increased hospitalization costs. Our study demonstrated that CF patients, especially undertreated patients, were associated with significantly higher treatment costs. Thereby we postulate that any strategy to prevent discordant treatment and CF is of great interest in terms of medical cost savings.

Assessment in CF patients was more severe than CS patients, similar to outcome of recent studies [18, 19], and laboratory and imaging findings in our data also supported these information. Direct etiology of CF related to CAP was defined as causes with the pulmonary infection and the systemic inflammatory response; and medical complications, such as cardiac arrhythmia, acute myocardial infarction, or the deterioration of comorbidities, were resulted from CAP-related systemic inflammatory response [17]. From our data, acute cardiac events and other organ dysfunctions were more common in CF group than CS group, suggesting complications were causes leading to failure.

Although the CURB-65 score may underestimate severity and mortality in the lower scoring patients, especially the elderly [20], in our study, the CURB-65 score was identified to be an independent risk factor for CF. In previous literatures, more data showed PSI or APACHE II score was independent factor associated with failure [4, 5, 21, 22]. Patients aged over 65 years old had more underlying comorbidity and poor outcome [10, 11, 15, 23]; meanwhile, data in our study revealed patients in CF group also had more underlying comorbidity than successfully treated patients. Aliberti et al. had found that a history of cardiac disease and cardiac events was related with clinical failure in CAP patients [17]; physicians should pay more attention to prevent cardiac complications as well as further investigations for these patients. Data from our study demonstrated acute heart failure and arrhythmias were the most common cardiac events during hospitalization, and a history of congestive heart failure was responsible for clinical failure related to elderly CAP patients. Additionally, we also found a history of bronchiectasis was a protective factor for clinical failure. We considered that was associated with administration of anti-pseudomonal β-lactam. In our recently published report [15], data showed that *Pseudomonas aeruginosa* was the most common pathogen in elderly CAP patients, overuse of anti-pseudomonal β-lactam, to some extent, may lead to a certain concordant treatment.

Discordant treatment accounted for more than 70% in CF group, significantly higher than CS group, and both were higher than data from Roson et al. [5]. In the prospective observational analysis, discordant therapy in early failure only constituted 30.8% [5]. Discordant treatment, especially overtreatment, is a very serious problem in China for CAP patients [15, 24]. In our study, some patients transferred from other hospitals or received antimicrobial treatment; therefore, the so-called initial antimicrobial treatment was not the really true initial treatment regimen. Part of the patients may have experienced clinical failure before admission. Wherefore a broad-spectrum antimicrobial treatment was prescribed after admission, resulting in overtreatment. Additionally, undertreatment was given for some patients due to inadequate assessment of severity and misjudgment of the pathogen.

Standardized antimicrobial treatment and a positive attitude toward guidelines is an urgent matter for all pneumology specialists. In our study, discordant treatment (undertreatment) was identified to be an independent risk factor for clinical failure, in accordance with previous data [4, 5, 25].

In this study, the multivariable logistic analysis of independent risk factors to predict failure also concluded PH<7.3, PaO_2_/FiO_2_<200 mmHg, hyponatremia and plural effusion. These factors were easily acceptable for clinicians, because they are already known to be associated with a poor prognosis and higher score on the PSI [26] or CURXO [27] or SMART-COP [28]. Studies from Menendez et al. [4], Hoogewerf et al. [9] and Aliberti et al. [17] had the similar conclusion that above variables were associated with clinical failure. A prospective multicenter cohort study performed in 1424 hospitalized patients from 15 Spanish hospitals revealed that leucopenia was associated with an almost four-fold higher risk of treatment failure [4]. Kolling and coworkers found that leucocytes had a beneficial effect on antimicrobial activity and modulation of the pro-inflammatory cytokine response in CAP [29]. In contrast, leukocytosis (WBC > 10,000/mm^3^) was confirmed to be a prognostic factor for failure in our data. In one CAPNETZ study about the inflammatory markers, they found WBC levels were associated with increasing CRB-65 score, while CRB-65 score was associated with short–term and long-term mortality [30]. Our data had discovered CURB-65 was a prognostic factor for failure; therefore leukocytosis was easily acceptable as a predictor of failure. Previous studies about elderly CAP patients revealed that male sex was associated with higher mortality [31] and higher rate of re-hospitalization [31, 32]. Interestingly, multivariable analysis in this study showed that male was a protective factor for failure. We surmise there are maybe differences in both the biological response to infection and therapeutic patterns of health care on sex.

Our study has some limitations. The present study was a retrospective observational study, to some degree, there was bias on data which may affect certain analysis. Additionally, the rate of clinical failure is underestimated than that in the real world. Our previous study had showed that 42.3% of the elderly CAP patients had a documented history of pre-hospital medication ^15^, and quite a few patients admitted to hospital was due to failure of pre-hospital antimicrobial treatment.

## Conclusions

This is the first attempt to explore the burden and prognostic factor for clinical failure of hospitalized elderly CAP patients in China. For country of aging society, it is important to recognize risk factors early, strengthen the awareness and concept of standardized treatment and reduce or avoid inappropriate treatment. Strategies for improved patient care should be implemented for specialists.

## Supplementary information

**Additional file 1 Table S1.** Univariate logistic regression analysis of prognostic factors for clinical failure associated with CAP. (*n* = 3011).

## Data Availability

All data generated or analyzed during this study are included in this published article and its supplementary information files.

## Abbreviations

LRTIs: Lower respiratory tract infections
CAP: Community-acquired pneumonia
CF: Clinical failure
ICU: Intensive care unit
CS: Clinical success
CIs: Confidence intervals
PSI: Pneumonia severity index
CHF: Congestive heart failure
HCAP: Healthcare-associated pneumonia
WBC: White blood cell
LOS: Length of stay
PaO_2_/FiO_2_: Arterial oxygen partial pressure / fraction of inspiration oxygen concentration
APACHE II: Acute physiology and chronic health evaluation II
CURXO: Confusion, urea nitrogen, respiratory rate, x-ray, PaO_2_
CAPNETZ: Community-acquired pneumonia network
