# Supplementary material for: Disease burden and prognostic factors for clinical failure in elderly community acquired pneumonia patients
Source: BMC Infect Dis. 2020 Sep 12;20:668. doi: 10.1186/s12879-020-05362-3 (PMC7486582; doi:10.1186/s12879-020-05362-3)
Supplement: Supplementary file 1 — Additional file 1 Table S1. Univariate logistic regression analysis of prognostic factors for clinical failure associated with CAP. (n = 3011). [file 12879_2020_5362_MOESM1_ESM.docx]

**Table S1. Univariate analysis of prognostic factors for clinical failure associated with CAP.**

**(n=3,011)**

|  | **TF**  **(n=395)** | **TS**  **(n=2616)** | ***P* value** |
| --- | --- | --- | --- |
| Age(years) | 79.3±7.87 | 77.1±7.3 | <0.001 |
| Male sex | 209(52.9) | 1431(54.7) | 0.505 |
| Aspiration | 79(20.0) | 234(8.9) | <0.001 |
| Long-term bedridden status | 74(18.7) | 185(7.1) | <0.001 |
| Underlying conditions |  |  |  |
| Cardiovascular disease | 268 (67.8) | 1607(61.4) | 0.014 |
| Hypertension | 215(54.4) | 1262(48.2) | 0.022 |
| Ischemic heart disease | 144(36.5) | 826(31.6) | 0.053 |
| Congestive heart failure | 46(11.6) | 150(5.7) | <0.001 |
| Chronic respiratory disease | 106(26.8) | 735(28.1) | 0. 603 |
| COPD | 77(18.5) | 482(18.4) | 0.611 |
| Bronchiectasis | 17(4.3) | 273(10.4) | <0.001 |
| Asthma | 16(4.1) | 139(5.3) | 0.291 |
| Cerebral vascular disease | 141(35.7) | 641(24.5) | <0.001 |
| Diabetes mellitus | 96(24.3) | 536(20.5) | 0.083 |
| Malignancy | 35(8.9) | 205(7.8) | 0.484 |
| Dementia | 11(2.8) | 44(1.7) | 0.131 |
| Chronic liver disease | 5(1.3) | 33(1.3) | 0.994 |
| HCAP | 135(34.2) | 385(14.7) | <0.001 |
| Immunocompromise | 18(4.6) | 52(2.0) | 0.002 |
| CURB-65 | 2.1±0.9 | 1.5±0.7 | <0.001 |
| Variables on admission |  |  |  |
| WBC>10000/mm^3^ | 172(45.1) | 762(29.7) | <0.001 |
| HCT＜30% | 86(22.9) | 310(12.4) | <0.001 |
| Cr>123.76umol/L | 68(18.2) | 217(8.7) | <0.001 |
| Glucose>14mmol/L | 19(5.5) | 68(2.8) | 0.010 |
| Sodium＜130 mmol/L | 55(14.7) | 141(5.6) | <0.001 |
| BUN>7mmol/L | 202(53.6) | 689(27.5) | <0.001 |
| PH＜7.3 | 24(7.7) | 21(1.3) | <0.001 |
| PaO_2_/FiO_2_＜200mmHg | 70(23.0) | 138(5.9) | ＜0.001 |
| Albumin＜25g/L | 57(15.7) | 154(6.2) | ＜0.001 |
| Multilobe infiltration | 1260(45.2) | 114(50.9) | 0.167 |
| Plural effusion | 662(23.8) | 87(38.8) | <0.001 |
| Antibiotic treatment |  |  |  |
| Adherent | 1081(38.8) | 66(29.5) | - |
| Undertreated | 463(16.6) | 71(31.7) | ＜0.001 |
| Overtreated | 1243(44.6) | 87(38.8) | ＜0.001 |

*Abbreviations*: CF, clinical failure; CS, clinical success; COPD, chronic obstructive pulmonary disease; HCAP, healthcare-associated pneumonia; WBC, white blood cell; HCT, hematocrit; Cr, Creatinine; BUN, blood urea nitrogen; PaO_2_/FiO_2, ,_ Partial arterial oxygen pressure/fraction of inspired oxygen.
